# Predictive factors of neurologic deterioration in patients with spontaneous cerebellar hemorrhage: a retrospective analysis

**DOI:** 10.1186/s12883-019-1312-8

**Published:** 2019-05-01

**Authors:** Yu-Ni Ho, Shih-Yuan Hsu, Yu-Tsai Lin, Fu-Chang Cheng, Yu-Jun Lin, Nai-Wen Tsai, Cheng-Hsien Lu, Hung-Chen Wang

**Affiliations:** 1 Departments of Emergency Medicine, Kaohsiung Chang Gung Memorial Hospital, Chang Gung University College of Medicine, Kaohsiung, Taiwan; 2 Departments of Neurosurgery, Kaohsiung Chang Gung Memorial Hospital, Chang Gung University College of Medicine, 123, Ta Pei Road, Niao Sung, Kaohsiung, Taiwan; 3 Departments of Otolaryngology, Kaohsiung Chang Gung Memorial Hospital, Chang Gung University College of Medicine, Kaohsiung, Taiwan; 4 Departments of Neurology Kaohsiung Chang Gung Memorial Hospital, Chang Gung University College of Medicine, Kaohsiung, Taiwan; 5 Department of Biological Science, National Sun Yat-Sen University, Kaohsiung, Taiwan

**Keywords:** Neurologic deterioration, ICH score, Spontaneous cerebellar hemorrhage, Risk factors

## Abstract

**Background:**

Cerebellar hemorrhage is a potentially life-threatening condition and neurologic deterioration during hospitalization could lead to severe disability and poor outcome. Finds out the factors influencing neurologic deterioration during hospitalization is essential for clinical decision-making.

**Methods:**

One hundred fifty-five consecutive patients who suffered a first spontaneous cerebellar hemorrhage (SCH) were evaluated in this 10-year retrospective study. This study aimed to identify potential clinical, radiological and clinical scales risk factors for neurologic deterioration during hospitalization and outcome at discharge.

**Results:**

Neurologic deterioration during hospitalization developed in 17.4% (27/155) of the patient cohort. Obliteration of basal cistern (p≦0.001) and hydrocephalus (p≦0.001) on initial brain computed tomography (CT), median Glasgow Coma Scale (GCS) score at presentation (p≦0.001) and median intracerebral hemorrhage (ICH) score (P≦0.001) on admission were significant factors associated with neurologic deterioration. Stepwise logistic regression analysis showed that patients with obliteration of basal cistern on initial brain CT scan had an odds ratio (OR) of 9.17 (*p* = 0.002; 95% confidence interval (CI): 0.026 to 0.455) adjusted risk of neurologic deterioration compared with those without obliteration of basal cistern. An increase of 1 point in the ICH score on admission would increase the neurologic deterioration rate by 83.2% (*p* = 0.010; 95% CI: 1.153 to 2.912). The ROC curves showed that the AUC for ICH score on presentation was 0.719 (*p* = 0.000; 95% CI: 0.613–0.826) and the cutoff value was 2.5 (sensitivity 80.5% and specificity 73.7%).

**Conclusion:**

Patients had obliteration of basal cistern on initial brain CT and ICH score greater or equal to 3 at admission implies a greater danger of neurologic deterioration during hospitalization. Cautious clinical assessments and repeated brain images study are mandatory for those high-risk patients to prevent neurologic deterioration during hospitalization.

## Background

Spontaneous cerebellar hemorrhage (SCH) account for represent 5 to 13% of all cases of spontaneous intracerebral hemorrhage and about 15% of cerebellar strokes [1–6]. It is often due to hypertension and the reported mortality rate within 6 months can reach 50%, and more than 60% of surviving patients had moderate or severe disability [6–8]. The prognostic risk factors of outcome in patients with SCH including hyperglycemia and platelet count at admission, a larger hematoma volumes or diameter, a lower Glasgow Coma Scale (GCS) at admission, and imaging findings that reveal the initial presence of hydrocephalus, intraventricular hemorrhage (IVH), the appearance of the fourth ventricle, or basal cistern obliteration have been reported in several studies [2, 6, 8–12].

Because of its unique neurological location near the brainstem, neurologic deterioration usually results from brain stem compression due to the direct mass effect of the haematoma and/ or the development of hydrocephalus [13]. However, here is still uncertainty about which risk factors significantly influence neurologic deterioration during hospitalization in patients with SCH.

This study aimed to identify potential clinical, radiological and clinical scales risk factors to predict neurologic deterioration during hospitalization and outcome at discharge in patients with SCH.

## Methods

### Study design

This is a single-centre retrospective study. Medical records were retrospectively reviewed using pre-existing standardized evaluation forms as well as brain computed tomography (CT) findings for patients with SCH admitted to the Department of Neurology or Neurosurgery in our tertiary academic centre from January 2005 to April 2015. The study approved by the Institutional Review Board (IRB)/Ethics Committee (Institutional Review Board numbers: 104-0985B).

### Clinical assessment

On admission, a detailed physical examination, the routine laboratory testing, and brain imaging were evaluated for all patients. The initial neurologic state was evaluated by the Glasgow Coma Scale (GCS). Then systolic blood pressure, diastolic blood pressure, heart rate, and body temperature were recorded immediately before brain computed tomography (CT) scanning. After CT scan, the ICH score was calculated by the all parameters [14]. The ICH score includes the following items: Glasgow Coma Scale score, age, infratentorial origin of ICH, cerebellar hemorrhage (CH) volume, and presence of IVH. The ICH Score was the sum of individual points assigned as follows: GCS score 3 to 4 (=2 points), 5 to 12 (=1), 13 to 15 (=0); age > =80 years yes (=1), no (=0); infratentorial origin yes (=1), no (=0); CH volume > =30 cm(3) (=1), < 30 cm(3) (=0); and intraventricular hemorrhage yes (=1), no (=0). The maximal score is 5 and the lowest score is 0.

**Fig. 1:**
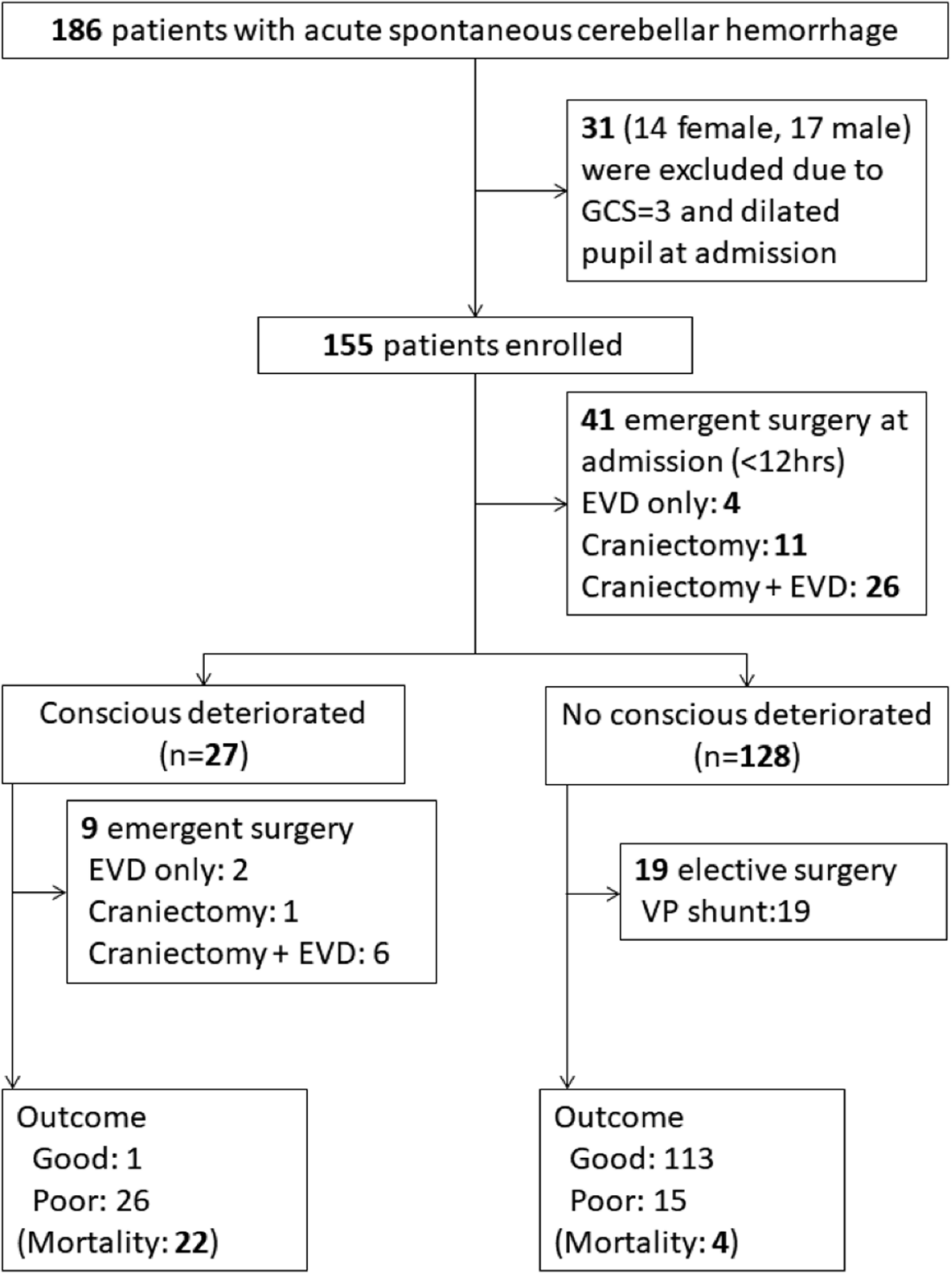
Flow diagram of patients with spontaneous cerebellar hemorrhage

Acute SCH was diagnosed by the clinical history and brain CT. Patients were excluded if they had: 1) non- spontaneous cerebellar hemorrhage, such as traumatic cerebellar hemorrhage; 2) SCH caused by a primary or secondary brain tumor, cavernomas, arteriovenous malformations or aneurysms, or hemorrhagic transformation of a cerebellar infarct; and 3) preexisting neurological conditions with various neurological deficits (such as stroke, head trauma, and hypoxic encephalopathy). From January 2005 to April 2015, a total of 186 patients with acute SCH were admitted to our hospital. This study is to evaluate neurologic deterioration during hospitalization, therefore, GCS =3 and no brain stem reflex on presentation was excluded. Finally, a total of 155 patients were enrolled in the studies (Fig. 1).

The SCH volumes were calculated the area occupied by the hyperdense hematoma with the ABC/2 formula in the initial brain CT. In the formula, A indicated the largest diameter of hemorrhage by CT, B indicated the largest diameter perpendicular to A, and C indicated the CT slice thickness multiplied by the number of CT slices of the hemorrhage [10, 15].

In the study, we determined the presence of hydrocephalus retrospectively by the dilated temporal horns of the ventricle. The image feature is consistent with obstructive hydrocephalus. [13, 16, 17]. The image feature of brain stem compression was judged by basal cistern obliteration in the brain CT [18, 19]. The fourth ventricle appearance was divided into 3 grades, according to Kirollos et al. study [1], as following: Grade 1, the 4th ventricle is in normal size and configuration, and located in the midline (if intraventricular hemorrhage is present, the cerebrospinal fluid is visible in the 4th ventricle); Grade 2, the 4th ventricle was partially compressed, distorted or shifting to the contralateral side (in cases of unilateral hematomas); Grade 3, the 4th ventricle is completely obliteration with shift distorting the brainstem anteriorly and obliteration of the prepontine space (even if partial compression of the 4th ventricle).

In the study, the neurosurgical interventions for management SCH were performed with three types: external ventricular drainage (EVD) only, midline suboccpital craniectomy for hematoma evacuation, and suboccpital craniotomy plus EVD.

Patients with neurologic deterioration during hospitalization was defined that patients presented the identified episodes of one or more of the following: 1) a spontaneous decrease in GCS motor scores of 2 points or more from the previous neurologic examination; 2) development of loss of pupillary reactivity 3) pupillary asymmetry greater than 1 mm. [20].

The patients were divided into two groups according to the discharge outcome: 1) The good outcome group with independent performance of daily activities (Glasgow Outcome scale (GOS) score = 4 or 5). 2) The poor outcome group with disability of daily living, vegetative states, or death (GOS score = 1, 2, or 3) [10]..

### Statistical analysis

The descriptive data were showed as both median and inter-quartile range (IQR). Categorical variables were accessed by the Chi-square test or Fisher’s exact test. The Mann-Whitney U test was used for the continuous variables analysis. The Spearman rank test was applied for correlation analysis for the relationship between age, GCS and laboratory data. Statistical significance was set at *p* < 0.05.

We used stepwise logistic regression analysis for evaluating the association between significant variables and patients with neurologic deterioration during hospitalization and outcome. ROC curve was generated to estimate an optimal cut-off value for ICH score on admission, and the area under ROC curve was measured. All statistical analyses were conducted using the SPSS software (IBM SPSS statistic version 22.0).

## Results

From January 2005 to April 2015, a total of 186 patients with SCH were admitted to our hospital. Thirty-one patients whose GCS was 3 and without pupil reflex at presentation were excluded. A total of 155 patients finally were enrolled in this study (Fig. 1).

### Baseline characteristics and clinical features

The baseline characteristics and clinical features are described in Table 1. A total of 155 patients were identified (84 males, 54.2%). The mean age was 66 years (IQR 18–87 years). Arterial hypertension was recognized as the most common underlying disease (122 patients, 78.7%) followed by diabetes mellitus (44 patients, 28.4%). The mean initial heat rate on arrive the hospital was 85 bpm (IQR 72-98 bpm) and the mean systolic arterial blood pressure on arrival in the hospital was 166 mmHg (IQR 142-195 mmHg). 26.5% (41/155) patients showed loss of consciousness at initial presentation. Otherwise, signs of SCH and increased intracranial pressure (e.g., unsteady gaits, nausea or vomiting) were presented at 90 patients (58.1%). The men symptoms duration was 1.5 (1.0–4.0) days.

**Table 1:** Characteristics of patients with spontaneous cerebellar hemorrhage

Parameter	Spontaneous cerebellar hemorrhage (*N* = 155)	%
Age (y), Median (range)	66 (18–87)	
Male	84	54.2
Initial heart rate, Median (IQR)	85 (72, 98)	
Initial systolic blood pressure (mmHg), Median (IQR)	166 (142, 195)	
Initial diastolic blood pressure (mmHg), Median (IQR)	90 (76, 101)	
Body temperature, Median (IQR)	36.5 (36, 36.9)	
Body mass index, Median (IQR)	23.7 (20.7, 25.8)	
GCS at admission		
4–8	34	21.9
9–12	18	11.6
13–15	103	66.5
Pupil had light reflex	130	83.9
Underlying diseases	–	
Hypertension	122	78.7
Diabetes mellitus	44	28.4
Liver cirrhosis	9	5.8
Atrial fibrillation	10	6.5
Coronary artery disease	5	3.2
Valvular heart disease	11	7.1
Old cerebral vascular accidents	30	19.4
End stage renal disease	10	6.5
Alcoholism	16	10.3
Smoking	19	12.3
Anti-coagulation medications		
Aspirin	8	5.2
Warfarin	2	1.3
Laboratory data at presentation, Median (IQR)	–	
WBC (X10^3^/mL)	9.9 (7.2, 13.2)	
Platelet counts (X10^3^/mL)	193 (143, 230)	
Prothrombin time (seconds)	10.4 (10, 11.1)	
International normalized ratio (INR)	1.0 (0.94, 1.08)	
Creatinine, mg/dL	0.98 (0.77, 1.27)	
Aspartate transaminase (AST), (IU/L)	30 (24, 43)	
Alanine transaminase (ALT), (IU/L)	23 (17, 35)	
Blood sugar, mmol/L	151 (129, 202)	
Signs and symptoms at onset	–	
Headache	60	38.7
Nausea, vomiting	90	58.1
Unsteady gait	20	12.9
Loss of consciousness	41	26.5
Limbs weakness	5	3.2
Symptoms duration (days)	1.5 (1.0, 4.0)	

*GCS* Glasgow coma score*, IQR* interquartile range

### Neuroradiological findings

Neuroradiological characteristics and neuro-surgical treatment at presentation were listed in Table 2. The median interval between hospital admission and the first brain CT was 45 min (IOR: 30–90 min). The median maximum hematoma dimension was 30.1 mm(IOR: 20–41.1 mm), corresponding to a median volume of 7.74 cm^3^ (IQR: 2.71–17.68 cm^3^) Hydrocephalus in initial image was presented in 69 patients (44.5%). The appearance of 4th ventricle compression was grade I in 30 (19.4%), grade II in 65 (41.9%), and grade III in 60 (38.7%) study sample patients. Eighty-four patients (54.2%) exhibited obliteration of the basal cistern.

**Table 2:** Radiological characteristics at presentation and neuro-surgical treatments

Parameter	Spontaneous cerebellar hemorrhage (*N* = 155)	%
Time to first brain images (minutes), Median (IQR)	45 (30, 90)	
Brain images findings at presentation	–	
Hematoma maximum dimensions (mm), Median (IQR)	30.1 (20, 41.1)	
Hematoma volume (mm3), Median (IQR)	7.74 (2.71, 17.68)	
Hematoma location		
Right hemisphere	69	44.5
Left hemisphere	44	28.4
Vermis	20	12.9
Involves more than two locations	22	14.2
Intraventricular hemorrhage grades		
Grade I	30	19.4
Grade II	65	41.9
Grade III	60	38.7
Hydrocephalus	69	44.5
Obliteration of basal cistern	84	54.2
Time to neuro-surgery (hours), Median (IQR)	2.46 (1.81, 9.0)	
Neuro-Surgical treatments		
EVD only	4	2.6
Suboccipital decompressive craniectomy	11	7.1
Suboccipital decompressive craniectomy + EVD	26	16.8

*EVD* External ventricular drainage*, IQR* interquartile range

Forty-one patients (26.5%) received neurosurgical intervention, including 11 patients (7.1%) with suboccpital decompressive craniectomy, 4 patients (2.6%) with EVD only, and 26 patients (16.8%)with both suboccpital decompressive craniectomy and EVD. There were 10 patients underwent the re-operative craniectomy. The median time between symptoms and surgery was 2.46 h (IQR: 1.81–9.0 h). The median time of neurologic deterioration after admission was 12.5 h (IQR: 6–72 h).

### Factors predict of neurologic deterioration during hospitalization and the outcome

Twenty-six (26/155, 16.8%) patients died during hospitalization. Twenty-three died of the hemorrhage. Three patients died of unrelated causes, one was aspiration pneumonia, one was massive GI bleeding, and the other one was uncontrolled liver cirrhosis. Factors predict of neurologic deterioration during hospitalization and the outcome in patients with SCH were listed in Table 3. In total 27 patients (17.4%) with SCH had neurologic deterioration during the hospitalization, 15 patients had spontaneous decrease in GCS motor score of 2 points or more from the previous examination, one had further loss of pupillary reactivity, three had development of pupillary asymmetry greater than 1 mm, five had both spontaneous decrease in GCS motor score of 2 points and further loss of pupillary reactivity, and three had both spontaneous decrease in GCS motor score of 2 points and development of pupillary asymmetry greater than 1 mm. Statistical analysis revealed significant associations of clinical feature and laboratory data at presentation are underlying disease with hypertension (*p* = 0.038), liver cirrhosis (*p* = 0.005), the initial presentation with loss of consciousness (*p* = 0.029), and elevated heart rate (*p* = 0.010). Statistical analysis revealed significant associations at initial neuro-image findings are median CH volume (p≦0.001), the presentation of obliteration of basal cistern (p≦0.001) and hydrocephalus (p≦0.001). But there is no significant statistical difference for patients with presentation of intraventricular hemorrhage in initial CT scan(*P* = 0.108). GCS and ICH score at presentation showed significant statistical difference between the two groups (both p≦0.001).

**Table 3:** Neurologic deterioration during hospitalization and outcome

	No neurologic deteriorated *N* = 128	Neurologic deteriorated *N* = 27	OR	*P*-Value
(1) Age (y), Median (range)	66.5 (18–87)	63 (20–86)		.288
(2) Male	70	14	1.099	.834
(3) Underlying diseases				
Hypertension	105	17	2.175	.038
Diabetes mellitus	37	7	1.133	.819
End-stage renal failure	6	4	.397	.073
Liver cirrhosis	5	4	.354	.050
Atrial fibrillation	8	2	.862	.686
Coronary artery disease	5	0	.820	.588
Valvular heart disease	8	3	.615	.409
Old cerebral vascular accidents	22	4	.806	.568
Alcoholism	12	4	.662	.483
Smoking	13	6	.489	.104
(4) Clinical feature at presentation				
Headache	46	14	.586	.134
Nausea, vomiting	77	13	1.491	.287
Unsteady gait	19	1	3.852	.202
Loss of consciousness	29	12	.450	.029
Limbs weakness	5	0	.820	.588
Pupil had light reflex	111	19	1.955	.240
(5) Laboratory data at presentation, Median (IQR)				
Heart rate, per minutes	81 (70, 95)	89 (84, 107)		.010
Systolic blood pressure, mmHg	164 (142, 193)	170 (143, 219)		.439
Diastolic blood pressure, mmHg	88 (77, 101)	90 (69, 101)		.839
WBC (X10^3^/mL)	9.6 (7.2, 13.1)	11 (7.9, 13.8)		.237
Platelet counts (X10^3^/mL)	197 (152, 230)	146 (81, 233)		.070
Partial thromboplastin time, seconds	10.3 (10.0, 11.0)	10.7 (9.8, 12.7)		.581
International normalized ratio	1.0 (0.95, 1.07)	1.02 (0.91, 1.24)		.978
Blood sugar, mmol/L	153 (128, 197)	151 (133, 246)		.655
(6) Brain Imagies Findings at presentation				
CH volume (mm3)	5.8 (1.9, 12.2)	20.2 (11.5, 35.1)		.000
Obliteration of basal cistern	60	24	.154	.000
Intraventricular hemorrhage	99	25	.331	.108
Hydrocephalus	48	21	.235	.000
(7) Median (IQR) GCS at presentation	15 (12, 15)	7 (5, 14)		.000
(8) Surgical intervention				
Extraventricular drainage (EVD)	4	2 (2^a^)		
Suboccipital craniectomy	6	6 (1^a^)		
Suboccipital craniectomy + EVD	17	15 (6^a^)		
Ventriculo-Peritoneal Shunt	19	6		
(9) ICH score, Median (IQR)	2 (2, 3)	3 (2, 4)		.000
(10) Outcome at discharged				
Good	113	1	.014	.000
Mortality	4	22	.046	.000

*GCS* Glasgow coma score*, OR* odds ratio*, SD* standard deviation*, IQR* interquartile range*, ICU* intensive care unit*,*

*CH* cerebellar hemorrhage

(^a^): emergent surgery while conscious deteriorated during hospitalization

Numerical variables were analyzed by Mann—Whitney U-test and Categorical variables were analyzed by chi-squared test and Fisher’s exact test

The functional outcome at discharged, the neurologic deterioration group showed poor outcome compared to the no neurologic deterioration group. Only 1 of 27 patients in neurologic deterioration group had good outcome, compared to 113 of 128 patients in no neurologic deterioration group (p≦0.001). Of these 27 patients had neurologic deterioration during hospitalization, 22 died during hospitalization. Of the five patients survived, only one had good outcome (GOS =5) with minor neurologic deficit (persistent headache, dizziness, and unsteady gait), two were vegetative state (GOS =2) and the other two were severe disability state (GOS =3) at discharged. Moreover, the SCH patients with neurologic deterioration during hospitalization revealed a specific high mortality rate during hospitalization. 22 of 27(81.4%) patients died during hospitalization in the neurologic deterioration group, compared to 4 of 128 (3.1%) patients died during hospitalization in no neurologic deterioration groups (P≦0.001).

Stepwise logistic regression analysis identified obliteran of basal cistern at initial brain CT and the higher ICH score as independent risk factors for neurologic deterioration during hospitalization. (*P* = 0.002 and *P* = 0.010, respectively) The adjusted risk of SCH patients with neurologic deterioration during hospitalization with obliteran of basal cistern at initial brain CT scan had odds ratio (OR) of 9.17 (95% confidence interval (CI): 0.026 to 0.455) compared with those without obliteran of basal cistern at initial brain CT scan. Furthermore, an increase of 1 point in ICH score on admission would increase the risks of neurologic deterioration during hospitalization rate by 83.2% (*p* = 0.010; 95% CI: 1.153 to 2.912).

To determine the relationship between ICH score on the initial presentation and risks of neurologic deterioration during hospitalization, the ROC curves were generated. The AUC for ICH score on presentation was 0.719 (p≦0.001, 95% CI: 0.613–0.826). The cutoff value of ICH score on presentation was 2.5 (sensitivity 80.5% and specificity 73.7%).

## Discussion

In our study, 17.4% patients with SCH had neurologic deterioration during hospitalization and most of these patients (26/27, 96.3%) had poor outcome. Arterial hypertension, initial loss of consciousness, the presentation of obliteran of basal cistern, hydrocephalus and CH volume in the initial brain CT, lower GCS and higher ICH score are statistically significant in patients with risks of neurologic deterioration. Although those factors are statistically significant in patients with neurologic deterioration, only obliteran of basal cistern at initial brain CT and the higher ICH score as independent risk factors (*p* = 0.002 and *p* = 0.010, respectively).

Several studies described the risks factors associated with neurologic deterioration in patients with spontaneous intracerebral hemorrhage. Flemming et.al [15] followed 61 patients with spontaneous supratentorial intracerebral hemorrhage and they demonstrated that the large volume lobar hematoma with consciousness disturbance (GCS < 14) and midline shift on CT are at risk for further deterioration. One recent meta-analysis [21] study mentioned the risks associated with early neurologic deterioration are hematoma volume, glucose concentration, fibrinogen concentration, and d-dimer concentration at hospital admission. Those studies were focused on supratentorial intracerebral hemorrhage. Many studies showed that initial impaired consciousness is a risk factor for poor outcome in patients with acute SCH [14, 22–24], however, there are limited studies specific focused on the risks of neurologic deterioration in patients with SCH or infratentorial hemorrhage.

In one previous study including 76 patients with SCH, the preoperative GCS and IVH were not prognostic factor for the patients’ outcome [13]. However, the GCS at admission was the risk factor for neurologic deterioration during hospitalization in our study. The difference between the two studies is the endpoint is different. Tsitsopoulos et.al study is to predict patient’s outcome, but our study is focused on neurological deterioration during hospitalization. However, most patients had neurological deterioration during hospitalization in our study had poor outcome.

Several studies have emphasized the initial radiologic features as risk factors for patient outcomes in the cases of SCH. The presence of hydrocephalus at admission, compression of the fourth ventricle, and obliteration of basal cisterns in neuroimages as indications for neurosurgical intervention and as prognostic factors in patients with SCH were mentioned in some of these studies [1, 4, 18, 19]. Indeed, the patients with neurologic deterioration during hospitalization is an another risk factor of prediction poor clinical outcome and risks of mortality in patients with SCH in this study. St. Louis et al. [25] followed 72 SCH patients and mentioned that acute hydrocephalus is the radiologic feature for predicting neurologic deterioration in patient with SCH. Our study also demonstrated the same result. In addition, the obliteration of basal cistern seen at initial brain CT may directly compress brainstem and cause neurologic deterioration. Previous studies also showed that the impaired conscious state seen in cerebellar hemorrhage can largely be attributed to the development of hydrocephalus, resulting in intracranial hypertension and/or direct brain stem compression [13, 24]. The both image feature imply the increase of posterior fossa intracranial pressure and impairment of the CSF circulation due to the cerebellar hematoma mass effect.

The ICH score was first introduced by Hemphill et al. [26] in 2001 and was a simple and reliable scale for predicting the 30-day mortality in patients with spontaneous intracerebral hemorrhage. The ICH score includes the following items: Glasgow Coma Scale score, age, infratentorial origin of ICH, CH volume, and presence of intraventricular hemorrhage. The maximal score is 5 and the lowest score is 0. Therefore, the ICH score is more representative than each of the above single factors. Several studies described the role of ICH score for predicting the outcome in patients with spontaneous intracranial hemorrhage [27–29]. In our study, an increase of 1 point in ICH score on admission would increase the risks of neurologic deterioration during hospitalization rate by 83.2%. ROC curve revealed the cutoff value of ICH score on presentation was 2.5 (sensitivity 80.5% and specificity 73.7%). Base on our study, because of risks of neurologic deterioration and high possibility of poor outcome, we suggest early cautious assessments for neurosurgical intervention for those SCH patients whose ICH score are greater or equal to 3.

In our study, the median time of neurologic deterioration after admission is 12.5 h. Of the 27 patients had neurological deterioration during hospitalization, only one had good outcome. For those high risk patients, cautious clinical assessment and repeated neuro-image study within the first 12 h after admission should be considered.

There are several limitations in our study. First, it is a retrospective review. Therefore, the decision for treatment of acute SCH, such as surgery, was individualized in each case, taking into account patient comorbidities. Second, neurologic deterioration can occur in both the acute stage and later stages during treatment. The findings may underestimate the “true” frequency of late neurologic deterioration in those patients who had been discharged. Thus, there is continued uncertainty in assessing the incidence of neurologic deterioration after SCH in non-selected patients. Lastly, this is a single hospital, small patient sample study. However, after excluding secondary cerebellar hemorrhage caused by trauma, tumors, cavernomas, arteriovenous malformations or aneurysms, hemorrhagic transformation of a cerebellar infarct, patients with “pure” acute SCH are not easy to collect. It needs prospective, multi-center research to clarify the causes of neurologic deterioration and outcome in future.

## Conclusions

In our study, 17.4% patients with SCH had neurologic deterioration during hospitalization and most of these patients (26/27, 96.3%) had poor outcome. Obliteration of basal cistern and hydrocephalus in the initial brain CT, lower GCS scale and higher ICH score on presentation implies a greater danger of neurologic deterioration during hospitalization. The median time of neurologic deterioration after admission was 12.5 h. Cautious clinical assessment, repeated neuro-image study within the first 12 h after admission and possibility of early neurosurgical intervention needed to be recognized by clinical physicians for high-risk patients, especially in those with ICH score greater or equal to 3, in order to early management the presence of neurologic deterioration in SCH patients.

## Abbreviations

CGS: Glasgow Coma Scale
CSF: Cerebrospinal fluid
CT: Computed tomography
EVD: External ventricular drainage
GOS: Glasgow Outcome scale
ICH score: Intracerebral hemorrhage score
IVH: Intraventricular hemorrhage
SCH: Spontaneous cerebellar hemorrhage
